# Localized Tail States and Electron Mobility in Amorphous ZnON Thin Film Transistors

**DOI:** 10.1038/srep13467

**Published:** 2015-08-25

**Authors:** Sungsik Lee, Arokia Nathan, Yan Ye, Yuzheng Guo, John Robertson

**Affiliations:** 1 Department of Engineering, University of Cambridge, Cambridge CB2 1PZ, United Kingdom; 2 Display and SunFab Solar Business Group, Applied Materials, Santa Clara, California 95054, USA

## Abstract

The density of localized tail states in amorphous ZnON (a-ZnON) thin film transistors (TFTs) is deduced from the measured current-voltage characteristics. The extracted values of tail state density at the conduction band minima (N_tc_) and its characteristic energy (kT_t_) are about 2 × 10^20^ cm^−3^eV^−1^ and 29 meV, respectively, suggesting trap-limited conduction prevails at room temperature. Based on trap-limited conduction theory where these tail state parameters are considered, electron mobility is accurately retrieved using a self-consistent extraction method along with the scaling factor ‘1/(α + 1)’ associated with trapping events at the localized tail states. Additionally, it is found that defects, e.g. oxygen and/or nitrogen vacancies, can be ionized under illumination with *hv* ≫ E_g_, leading to very mild persistent photoconductivity (PPC) in a-ZnON TFTs.

Oxide semiconductors shows great promise as a high mobility channel layer in thin film transistors (TFT) fabricated at low or even room temperatures^1^,^2^. A classical oxide material is ZnO, which has mostly a poly-crystalline (pc) structure, i.e. pc-ZnO, suggesting the presence of potential barriers at the grain boundaries, which lowers the mobility (see Fig. 1a)^3^,^4^. To suppress these potential barriers, metal cations, such as Ga and In, were incorporated into the binary system, thereby stabilizing the amorphous phase over the crystalline phase. But the addition of Ga and In introduces compositional disorder, and these cation states create potential fluctuations in the conduction band minima (E_C_)^5^,^6^. Although this tends to reduce the overall electron mobility as shown in Fig. 1b, the approach has been successful due to the low temperature processability and high mobility in the amorphous phase^1^,^7^.

As another approach for high mobility and optical stability in oxide semiconductors, anions, instead of cations, can be added^8^. For example, flourine (F) can be used for n-type doping of SnO_2_. However, F does not affect electron mobility as it is an anion, which puts disorder into the valence band not the conduction band^9^. As another example, when nitrogen (N) is incorporated into ZnO, this does not cause p-type doping but it forms an amorphous alloy, where the anion site disorder stabilizes the amorphous phase, making amorphous ZnON (a-ZnON). Again, this does not lower the electron mobility because the disorder is in valence band states, and not the conduction band states. Another important effect is that the N 2p orbital lies higher than the O 2p orbital, so that the new N 2p states raise the valence band maxima (VBM) above its energy in ZnO^9^. It is known that the photo-induced instabilities of ZnO and IGZO are related to states due to O vacancies and interstitials that lie in the lower band gap region. These states are now covered up by the higher VBM states, so they can no longer give rise to such instabilities and persistent photoconductivity (PPC)^10^,^11^, which is a strong benefit for the N containing materials.

According to the recent literature^11^, the electron effective mass of a-ZnON (~0.19 m_0_) can be larger than that of crystalline ZnN (i.e. c-ZnN) and smaller compared to ZnO films. This may be explained with a disorder of a-ZnON, especially in amorphous phase. Indeed, the conduction band minima (CBM) of a-ZnON is also composed of both Zn 4 s and N 2p, similar to c-ZnN^12^,^13^. Here, N 2p is sensitive to bonding angle tilt in amorphous phase, thus high disorder in a-ZnON, although Zn 4 s is spherical and less sensitive to bonding angle disorder. This can yield a high density of localized tail states near the CBM in a-ZnON (see Fig. 1c). For a TFT with a-ZnON channel, the field-effect mobility is strongly affected by the presence of the localized tail states. This can be explained with trap-limited conduction theory (i.e. multiple trapping and thermal release events) (see Fig. 1c,d)^5^,^14^. One of the ways to reduce the localized tail state density is by thermal annealing, resulting in higher mobility (up to 110 cm^2^/V-s) reported in Ref. 8.

In this paper, we extract the density of localized tail states in a-ZnON TFTs, using current-voltage characteristics of the device. The extracted values of tail state density at the CBM (N_tc_) and characteristic energy (kT_t_) are about 2 × 10^20^ cm^−3^eV^−1^ and 29 meV, respectively, thus kT_t_ > kT (i.e. thermal energy) at T = 300K. This implies that trap-limited conduction is dominant at room temperature. In addition, it is found that the extracted field-effect mobility and its gate voltage-dependence are strongly dependent on the pre-factor 1/(α + 1), where α = 2(kT_t_/kT-1) for kT_t_ > kT. Here, we derived a more accurate field-effect mobility expression, using the proposed self-consistent extraction method. Also, a weak persistent photoconductivity in a-ZnON TFTs with Mo electrode is observed under visible light illumination, suggesting ionization of defects, such as oxygen and/or nitrogen vacancies, located near the VBM. To check the effect of electrode on leakage current and PPC, we replaced the Mo with Cr for electrodes. It is found that the leakage current slightly increases, but the effect of illumination is maintained, suggesting the PPC is arising from a change in the intrinsic property of the channel layer.

## **R**esults and Discussion

### Localized Tail States

Since the current-voltage (I-V) characteristics of the TFT are largely determined by the density of localized states, e.g. tail states in the channel layer, the density of localized states can be retrieved from the measured terminal characteristics^15^,^16^,^17^. As a first step, the free carrier density (n_free_) is extracted from the measured I-V characteristics. Note that linear characteristics of the drain current vs. gate voltage (I_DS_-V_GS_) are required rather than saturation regime characteristics^16^,^17^,
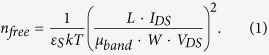
Here, μ_band_ is the band mobility of the ZnON layer (note that this is a constant and the main unknown), ε_S_ the permittivity of a-ZnON (which is about 11ε_0_, where ε_0_ is vacuum permittivity), kT the thermal energy, W the channel width, L the channel length, and V_DS_ the drain voltage. Also, the carrier density of the free carriers (n_free_) and trapped carriers (n_trap_) at tail states can be derived with Poisson’s Equation along with Gauss’s law as follows,
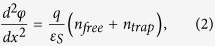

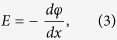
where x is the distance from the front channel interface along the channel depth, and φ the channel potential along x. Based on Equations (2) & (3), the carrier densities can be connected to gate voltage using a charge balance Equation: E(x = 0) = C_ox_(V_GS_ − V_Te_). Here, C_ox_ is the gate-insulator capacitance and V_Te_ is an effective threshold voltage which can be represented as [1/V_GS_ + 1/V_T_]^−1^ for V_GS_ > 0 and V_T_ > 0, where V_T_ is a threshold voltage to be extracted with a linear extrapolation at a linear regime. This yields the following,
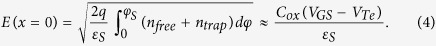


Squaring Equation (4) and taking its first derivative with respect to the surface potential φ(x = 0) = φ_S_, n_trap_ can be obtained as,
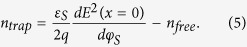


Based on Equations (1), (4), and (5), the density of tail states (N_tail_(E)) can be given as the first derivative of Equation (5),
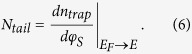
Here, qφ_S_ = E_F_ − E_F0_ where E_F0_ is the Fermi level at flat band (see the Supplementary Information for more detailed derivation procedure).

**Figure 2:**
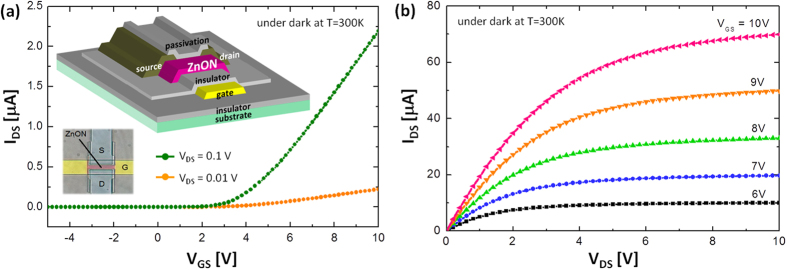
(a) Linear I_DS_-V_GS_ characteristics measured at a small V_DS_ (0.01 V and 0.1 V). Inset: Schematic 3-D view of the fabricated a-ZnON TFTs along with this micro-photo. Here, the gate insulator is Si_3_N_4_, stacked layer of SiO_2_ and Si_3_N_4_ is used as Passivation, and Mo and Cr are used for source, drain, and gate terminals in two different devices, respectively. (**b**) Measured output characteristics, i.e. I_DS_-V_DS_, of the examined TFTs for different V_GS_.

**Figure 3:**
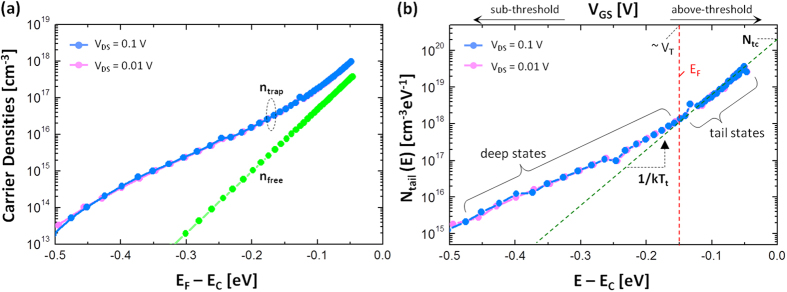
(a) Extracted carrier densities (n_free_ and n_trap_) and (**b**) density of tail states denoted as N_tail_(E). The extracted N_tail_(E) for different V_DS_ is similar to each other, suggesting that the extraction method is almost independent on V_DS_ when V_DS_ is small enough. This also implies that contact resistance effects are negligible at small V_DS_, e.g. 0.1 V and 0.01 V.

Based on the above extraction method, the density of localized tail states was retrieved using a-ZnON TFTs with W = 50 μm and L = 10 μm. Here, the linear I_DS_ − V_GS_ characteristics were measured at a small V_DS_ (e.g. 0.01 V and 0.1 V), as seen in Fig. 2(a,b) shows output characteristics for different V_GS_ (see also Figure S2 in the Supplementary Information). The basic parameter to be extracted from each I-V curve is V_T_. The V_T_ values of these two cases (V_DS_ = 0.01 V and 0.1 V) are 4.4 V and 4.3 V, respectively. The other unknown parameters, μ_band_ and C_ox_ are 110 cm^2^/V-s and 19 nF/cm^2^, respectively^8^. Here, μ_band_ can be the maximum achievable mobility and the C_ox_ value was calculated using a thickness of 300 nm and measured permittivity of ~6.5ε_0_ for the gate insulator Si_3_N_4_. Using these parameters and Equations (1, 2, 3, 4, 5, 6), the carrier densities (n_free_ and n_trap_) and density of tail states (N_tail_(E)) were extracted as seen in Fig. 3a,b, respectively. As indicated in Fig. 3b, the tail states (i.e. gap states near the E_C_) can be approximated as an exponential distribution^14^,^15^,^16^,
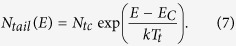
Here, N_tc_ is the tail state density at E = E_C_ (i.e. conduction band minima), and kT_t_ is the characteristic energy of the tail state. Applying Equation (7) into the plot shown in Fig. 3b, N_tc_ and kT_t_ values were extracted as 2 × 10^20^ cm^−3^eV^−1^ and 29 meV, respectively.

### Electron Mobility

**Figure 4:**
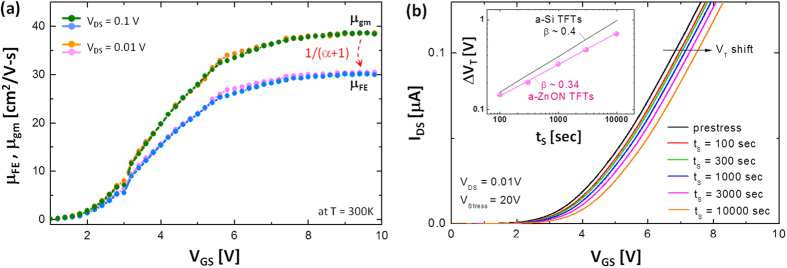
(a) Field effect mobility (i.e. gate voltage dependent mobility) as a function of V_GS_ for different V_DS_: 0.1 V, 0.01 V. Here, μ_FE_ is shown from using Equation (19) in comparison with μ_gm_ calculated by the conventional way with transconductance (g_m_). (**b**) I_DS_-V_GS_ characteristics measured after applying 20 V stress for each stress period (t_S_). Here, the gate voltage applied (V_Stress_) is 20 V (Inset: retrieved ∆V_T_ vs. t_S_).

To incorporate the effect of tail states into the field effect mobility (μ_FE_), the trap-limited conduction theory is employed,
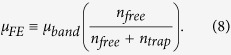
Here, n_free_ (with Boltzmann’s approximation for |E_F_ − E_C_| > kT) and n_trap_ (for kT_t_ > kT) are represented analytically as a function of Fermi level (E_F_)^16^,^17^, respectively, as follows,
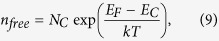

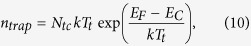
where N_C_ is an effective free carrier density [cm^−3^]. Note that the N_C_ value of ZnON is about 2 × 10^18^ cm^−3^, which is calculated with an electron effective mass ~0.2 m_0_^10^,^11^, where m_0_ is the electronic rest mass. With Equations (9) & (10), Equation (8) can be rewritten as a function of E_F_, assuming n_free_ < n_trap_,
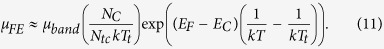
Here, E_F_ − E_C_ (=E_F0_ + qφ_S_ − E_C_) can be given as a solution of Equation (4), replacing V_Te_ by V_T_ just for the above-threshold regime,
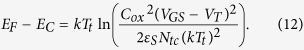


Using Equations (11) & (12), Equation (11) can be represented as a function of V_GS_, following a power law,
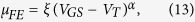

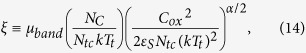
where α = 2(kT_t_/kT − 1). Note that ξ is a prefactor independent on V_GS_. As seen in Equation (13), the field-effect mobility (μ_FE_) is a function of V_GS_, and linked with localized tail states in terms of the exponent (α) and constant (ξ) in the power law. Along with Equation (13), the current-voltage relation can be derived based on a drift transport equation, and approximated with the condition, V_GS_ − V_T_  ≫ V_DS_ (i.e. linear approximation), as follows,
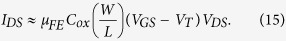


Due to the presence of the exponent (α) and gate-voltage dependence in the μ_FE_ expression seen in Equation (13), the first derivative of Equation (15) with respect to V_GS_ (i.e. transconductance (g_m_)) is given as follows,
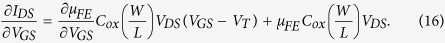


Note that the first term of Equation (16) cannot be zero since μ_FE_ is a function of V_GS_. So, the conventional way to get μ_FE_ seems to be insufficient and inconsistent. In Equation (16), the first derivative of μ_FE_ is shown and can be expanded with Equation (13), as follows,
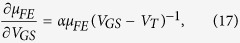


With Equation (17), Equation (16) can be rewritten as follows,
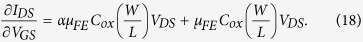


From Equation (18), μ_FE_ is now given as follows,
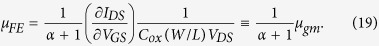


As can be seen in Equation (19), due to the presence of α associated with localized tail states, the mobility can be reduced by the ratio of 1/(α + 1). Here, we defined the parameter ‘1/(α + 1)’ as the mobility scaling factor due to trapping events in the localized tail states. So, the conventional way yields an over-estimated value and hence an inconsistency. So, we believe that Equation (19) provides a more accurate μ_FE_(V_GS_) while capturing the effects of localized tail states with the parameter α. Using Equation (19), μ_FE_(V_GS_) was extracted using the retrieved value of kT_t_ seen in Table 1. In addition, the following equation can also be defined to explain the portion of trapping (ψ) as,
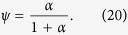


This can be used as a measure of how trapping significantly affects the electron mobility. As shown in Fig. 4(a), μ_FE_ (at V_GS_ = 9 V) is about 30.2 cm^2^/V-s for V_DS_ = 0.01 V which is reduced by 21% (i.e. ψ = 0.21) compared to the conventional extraction route where μ_FE_ is considered as a constant (38.4 cm^2^/V-s without considering α), i.e. μ_gm_. Note that we also tested the proposed mobility extraction method for V_DS_ = 0.1 V. It is found that there is a discrepancy less than 1% compared to the case of V_DS_ = 0.01 V, as summarized in Table 1. Here, we believe that the case of V_DS_ = 0.01 V provides higher accuracy compared to V_DS_ = 0.1 V since a smaller V_DS_ is always better to satisfy the assumption that V_GS_ − V_T_ ≫ V_DS_, relating to Equation (15).

The band tail states not only affect the electron mobility but also affect bias instability. Indeed, it is known that the density of band tail states determines the rate of instability creation, e.g. threshold voltage shift (ΔV_T_). The ΔV_T_ as a function of time (t) is defined by the following relation^18^,
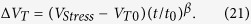
Here, V_Stress_ is the bias stress, V_T0_ the pre-stress threshold voltage (~4.3 V), and t_0_ the characteristic time constant. In particular, β is a power-law exponent, which is proportional to the tail state density (N_tc_, cm^−3^eV^−1^), So the following relation can be deduced,
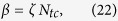
where ξ is a constant in the dimension of cm^3^eV. To extract the values of β and t_0_, the I-V characteristics were measured after applying 20 V bias stress for each stress period, as seen in Fig. 4b. Based on this, the ΔV_T_ as a function of time is retrieved, as seen in the inset of Fig. 4b, yielding β ~ 0.34 and t_0_ ~ 10^8^ sec. Here, it is found that the value of β is smaller compared to a-Si TFTs ~ 0.4^18^. As can be seen in Equation (22), this can be explained with a smaller N_tc_ of a-ZnON TFTs ~ 2 × 10^20^ cm^−3^/eV compared to a-Si TFTs ~ 10^22^ cm^−3^/eV^19^.

### Persistent Photoconductivity

**Figure 5:**
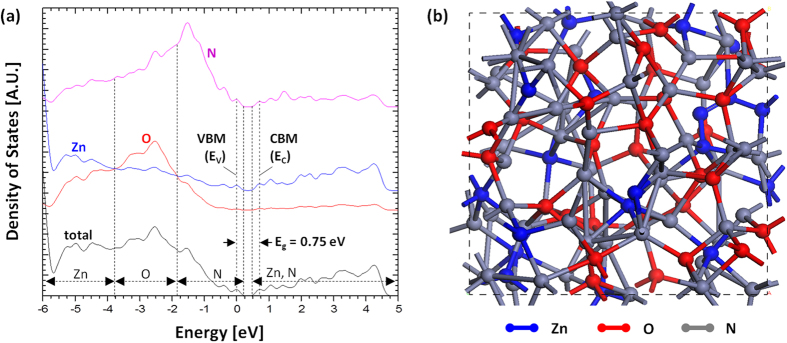
(a) Computed total electronic density of states (DOS) and projected DOS (PDOS) for the Zn, O, and N atoms in a-ZnON. The VBM energy level is set to 0 eV, and retrieved band-gap energy (E_g_) is about 0.75 eV as indicated. (**b**) The model unit cell of a-ZnON obtained from the melt-quench simulations.

**Figure 6:**
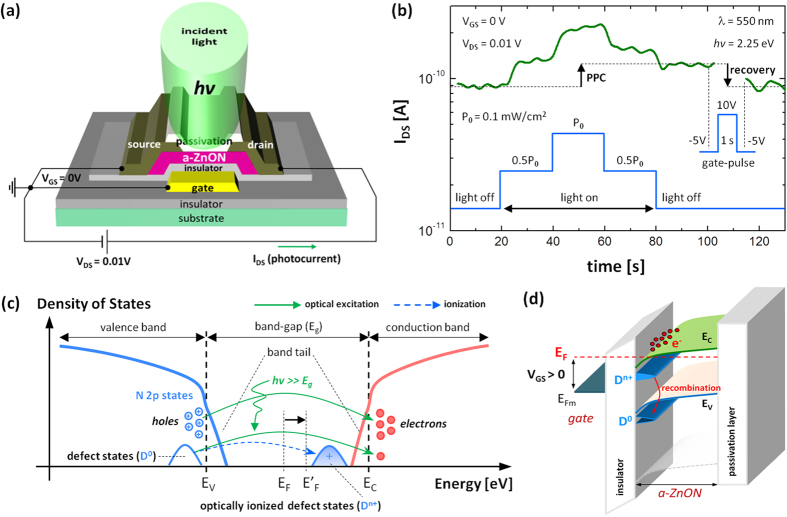
(a) Schematic 3-D view of the transistor examined with the light illumination and DC bias on V_DS_. (**b**) Measured drain current (I_DS_) of the examined device with Mo electrode as a function of time. Here, light pulse is applied as described in the inset. (**c**) Possible density of states picture with respect to optical carrier generation mechanisms and PPC. Here, the generated electrons are denoted as ‘e’, E_F_ and E’_F_ denote Fermi levels before and after illumination, respectively, and E_V_ is the valence band maxima. (**d**) Schematic 3-D band diagram along the channel depth. Here, we show the effect of the positive gate bias on the recombination of ionized deficiency defects with induced electrons.

Additionally, we performed computations of the density of states (DOS) in a-ZnON. Figure 5 shows the computed total DOS and projected DOS (PDOS) onto Zn, O, and N atoms for a-ZnON. Note that the fabricated a-ZnON film composition has been measured with the Rutherford backscattering spectrometry (RBS) (see the Supplementary Information). As shown in Fig. 5a, it is clear that the N forms the VBM, and is located from −1.8 eV to 0 eV. This implies that oxygen defects (e.g. vacancies and interstitials), viewed as the origin of the PPC, are filled with N. Also, the band-gap is about 0.75 eV, as indicated in Fig. 5, suggesting that photon energies in the range from 0.75 eV ~ 2.55 eV may not give rise to the PPC in a-ZnON. To check this, we measured the drain current as a function of time (i.e. I_DS_-time plot) when V_GS_ = 0 V and V_DS_ = 0.01 V under illumination with 550 nm wavelength light (equivalent to 2.25 eV photon energy, *hv*), as seen in Fig. 6a. Here, the maximum optical power P_0_ is ~0.1 mW/cm^2^. As shown in Fig. 6b, the drain current (~2.3 × 10^−10^ A) under P_0_ is increased by 2 times more than that (~1.2 × 10^−10^ A) under 0.5P_0_. This suggests that the drain current under illumination is associated with excess carrier generation, which is linearly proportional to incident optical power, rather than electron trapping into gate insulator. After removal of illumination (after t = 80 s), the drain current is almost recovered, thus the examined a-ZnON is optically very stable. However, we find a small difference (i.e. I_PPC_ ~ 3.4 × 10^−11^ A) between the initial (I_DS_ ~ 9.1 × 10^−11^ A) and final (I_DS_ ~ 1.25 × 10^−10^ A) stages. Thus, persistent photoconductivity (PPC) still exists, albeit mild as seen in Fig. 6b, implying that some of the excess electrons are generated from optically irreversible states. So, the PPC may be associated with some of the unfilled oxygen defects (O_D_). Besides oxygen-related defects, we also consider nitrogen-related defects (N_D_), such as nitrogen vacancies (N_V_), which exist especially at the vicinity of the valence band maxima^20^,^21^,^22^. Under illumination, they can also be ionized as N_V_^0^ → N_V_^n+^ + ne^–^, where n is an integer, e.g. 1 ~ 3^20^,^22^. Since these constitute negative U-defects, the ionization process is irreversible even after removal of light, thus giving rise to the mild PPC^22^. These defects, collectively denoted as D^0^, can be ionized under illumination (D^0^ → D^n+^ + ne^−^), relocating to the vicinity of the E_C_^10^,^11^,^20^,^21^,^22^,^23^,^24^,^25^. This maintains the increased Fermi level (E_F_) even after illumination, as described in Fig. 6c. Since we have a very mild PPC (see Fig. 6b), we should assume that the density of defects, including oxygen and nitrogen vacancies, is small, implying that the photocurrent under illumination mostly consists of electrons generated from the N 2p states in the valence band. In order to estimate the number of the ionized deficiency defects (N_idd_, cm^−2^), we may use the following relation,
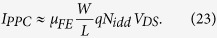


Using the values of the parameters extracted in the previous sections (e.g. I_PPC_ = 3.4 × 10^−11^ A, μ_FE_ = 30.2 cm^2^/V-s, W/L = 5, V_DS_ = 0.01 V), the N_idd_ is retrieved as 1.43 × 10^8^ cm^−2^. To electrically remove these ionized defects, we employed a positive gate-pulse scheme^25^, as seen in Fig. 6b. This yields a fully recovered current level (I_DS_ ~ 9.2 × 10^−11^ A) which is similar to the current level before illumination. Hence, it is suggested that these ionized defects (D^n+^) were eliminated with the forced recombination with the electrons (ne^−^) induced during the positive gate-pulse width (+ 10 V), i.e. D^n+^+ne^−^ → D^0^, as described in Fig. 6d. Here, the number of induced electrons (i.e. N_e_ ≈ C_ox_ × (10 − V_T_)/q ≈ 6.7 × 10^11^ cm^−2^) are much more than the N_idd_ ~ 1.43 × 10^8^ cm^−2^, thus it is enough for a full recombination.

**Figure 7:**
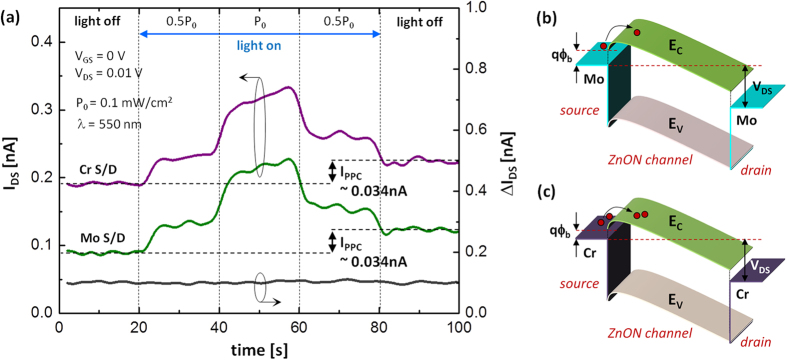
(a) Measured IDS vs. time for two devices with different metal electrodes, e.g. Mo and Cr, respectively. Also, the current difference is shown on y-axis on the right hand side. Horizontal band diagrams for the cases of (**b**) Mo and (**c**) Cr electrodes, respectively.

In addition, we replaced Mo with Cr for electrodes to check effects of metal on leakage current and PPC. Figure 7a shows the measured I_DS_ vs. time for two devices with different metal electrodes, e.g. Mo and Cr, respectively. It is found that there is a small current difference before and after illumination for each case. And the difference (i.e. I_PPC_) is almost the same as 0.034 nA. This implies that the choice of electrode metal doesn’t affect the PPC. And the current difference between the Mo and Cr cases (ΔI_DS_) is shown on the right-hand-side y-axis. It is found that this current difference is always approximately 0.1 nA, suggesting that the leakage current is changed globally regardless of illumination and PPC. This can be explained with the reduced barrier height at source side (qϕ_b_) due to a smaller work-function of Cr (~4.5 eV) compared to Mo (~4.6 eV), as shown in Fig. 7b,c. These results indicate that the choice of metal for electrodes does not affect the PPC.

## Conclusions

In conclusion, the density of localized tail states in ZnON thin film transistors (TFTs) has been extracted using current-voltage characteristics of the TFTs. The extracted values of N_tc_ and kT_t_ are about 2 × 10^20^ cm^−3^eV^−1^ and 29 meV, respectively. Considering trap-limited conduction theory, the field-effect mobility expression has been derived and shown to be represented in terms of tail state parameters. In particular, the exponent (α) has been strongly connected to the mobility through kT_t_ which is a key measure of the degree of the disorder of the channel layer. This suggests that a reduction of kT_t_ is needed to achieve higher mobility. Additionally, it has been revealed that the examined ZnON is optically very stable showing only weak PPC which is thought to be arising from ionization of defects, such as oxygen and/or nitrogen vacancies, located in vicinity of the valence band maxima. This happens equivalently in both ZnON TFTs with Mo and Cu electrodes, suggesting the PPC is associated with a change of the intrinsic property of the channel.

## Additional Information

**How to cite this article**: Lee, S. *et al.* Localized Tail States and Electron Mobility in Amorphous ZnON Thin Film Transistors. *Sci. Rep.* **5**, 13467; doi: 10.1038/srep13467 (2015).

## Supplementary Material

Supplementary Information

## Figures and Tables

**Figure 1:**
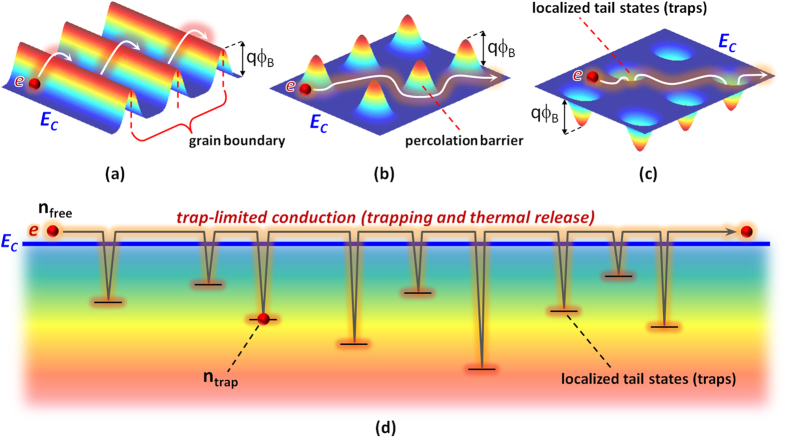
Schematic profiles of the conduction band minima (E_C_) in (**a**) poly-crystalline ZnO, (**b**) amorphous InGaZnO, and (**c**) amorphous ZnON, respectively. Here, ‘e’ denotes a free electron released into conduction band. In (**a**), φ_B_ is the potential barrier height at the grain boundary in poly-crystalline ZnO. In (**b**), φ_B_ is the potential barrier height due to compositional disorder in amorphous InGaZnO. (**d**) Schematic diagram to describe trap-limited conduction associated with the localized tail states. Here, n_free_ and n_trap_ denote free and trapped carrier densities at band tail states, respectively.

**Table 1:** Summary of Extracted Parameters for a different V_DS_.

Parameters	V_DS_	
	0.01 V	0.1 V
N_tc_ [cm^−3^eV^−1^]	2.15 × 10^20^	2.17 × 10^20^
kT_t_ [meV]	29.1	29.2
1/(α + 1)	0.79	0.78
ψ	0.21	0.22
μ_gm_ [cm^2^/V-s] (peak)	38.4	38.6
μ_FE_ [cm^2^/V-s] (peak)	30.2	30.1
