# Supplementary material for: Localized Tail States and Electron Mobility in Amorphous ZnON Thin Film Transistors
Source: Sci Rep. 2015 Aug 25;5:13467. doi: 10.1038/srep13467 (PMC4548246; doi:10.1038/srep13467)
Supplement: Supplementary Information [file srep13467-s1.pdf]

Supplementary Information

Localized Tail States and Electron Mobility in Amorphous ZnON Thin Film Transistors

Sungsik Lee[1], Arokia Nathan[1]*, Yan Ye[2]†, Yuzheng Guo[1], and John Robertson[1]‡

1. Department of Engineering, University of Cambridge, Cambridge CB2 1PZ, United Kingdom
*E-mail: an299@cam.ac.uk, Phone: +44-1223-748302, Fax: +44-1223-748322
‡E-mail: jr214@cam.ac.uk, Phone: +44-1223-748334, Fax: +44-1223- 748348

2. Display and SunFab Solar Business Group, Applied Materials, Santa Clara, California 95054, USA
†E-mail: yanye7@gmail.com, Phone: +1-408-748-5227, Fax: +1-312-601-4348
(*,†,‡ : Corresponding Authors)

List of Information:

S1. RBS Measurement Results for ZnON films

S2. Input and Output Characteristics of the Fabricated ZnON TFTs

S3. Detailed Derivation Procedure of Density of Localized Tail States

S1. RBS Measurement Results for ZnON films

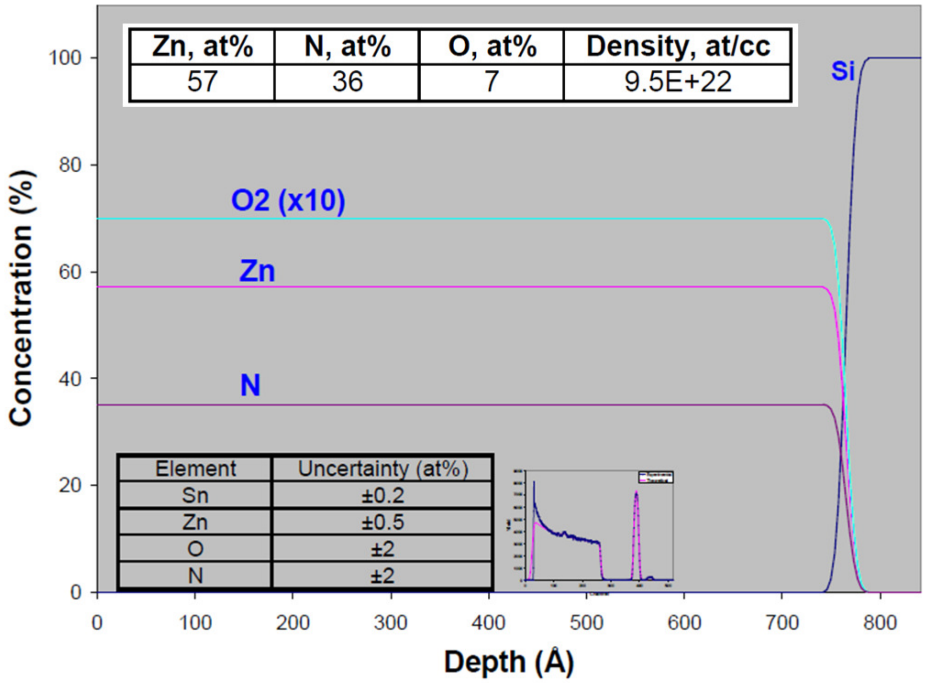

Figure S1: RBS Measurement Results for ZnON films. Here, N at% is about 2.6% higher than stoichiometric concentration (measurement uncertianty is +2 at%), if assuming 1:1 for ZnO and 3:2 for Zn3N2. Other chemical bonds may exist in the film.

S2. Input and Output Characteristics of the Fabricated ZnON TFTs

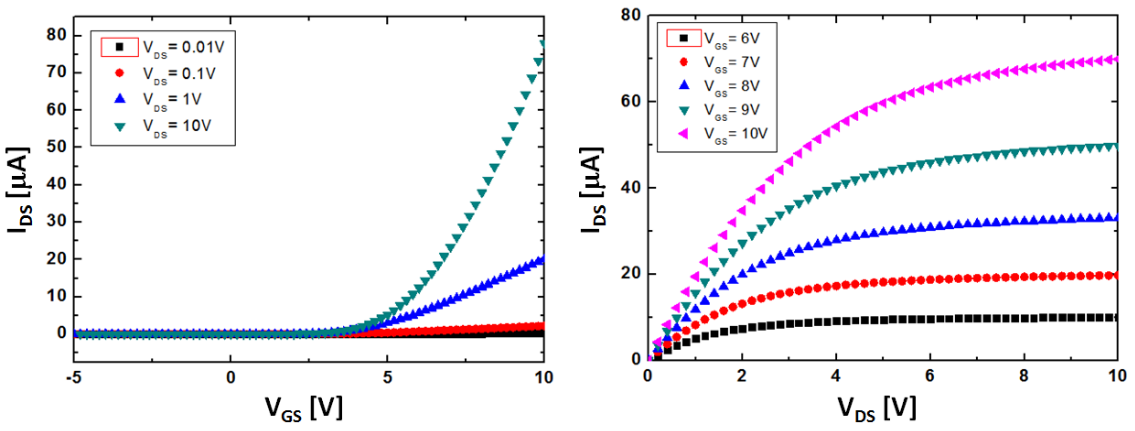

(a) **(b)**

Figure S2: (a) Input and (b) Output Characteristics of the Fabricated ZnON TFTs

S3. Detailed Derivation Procedure of Density of Localized Tail States

S3.1. Relationship between Surface Potential and Gate Voltage

S3.1.1. 2D Poisson's Equation and Its Approximation

Fig.S3.1 depicts the schematic cross-section of a bottom gate TFT. Based on Fig.S3.1, the line integral for the electric field yields a two-dimensional Poisson's equation in integral form,

$$\oint_C \vec{E}\cdot\vec{dl} = \int_0^x E_y dx - \int_0^y E_x dy = -\frac{q}{\varepsilon_S}\int_0^x\int_0^y \left(n_{free}+n_{trap}\right)dydx\,. \qquad \text{(S3-1)}$$

Here, the electric field is perpendicular to each path (initial conditions: E_x(x=0)=0 & E_y(y=t_S)=0). While satisfying $|E_y| >> |E_x|$ for $V_{GS}>>V_{DS}$, Eq.(S3-1) can be approximated with $E_y= -d\varphi/dy$ to,

$$E_y(y=0)=\sqrt{\frac{2q}{\varepsilon_S}\int_0^{\varphi_S}[n_{free}(\varphi)+n_{trap}(\varphi)]d\varphi} \equiv E_S\,, \qquad \text{(S3-2)}$$

where φ_S is an effective surface potential at y=0. Here, the surface electric field E_S can also be defined as $E_S= (Q_{free}+Q_{trap})/\varepsilon_S$ by Gauss's law (where Q_{free} is free charge and Q_{trap} is trapped charge at localized states including tail and deep states). Considering interface state charge Q_{it}, $Q_{free}+Q_{trap}$ can be obtained by the charge neutrality condition at the interface, $C_{ox}(V_{GS}-V_{FB}-\varphi_S)=\varepsilon_S E_S+Q_{it}$ with $Q_{it}=C_{it}\varphi_S$ (where C_{it} is the mean value of interface state capacitance and V_{FB} is an effective flat-band voltage, while considering work-function difference (ϕ_{ms}) as well as a trapped charge (Q_{ox}) in a gate-insulator, as $V_{FB}=\phi_{ms}-Q_{ox}/C_{ox}$). This yields,

$$Q_{free}+Q_{trap}=C_{ox}\left(V_{GS}-V_{FB}-\left(1+\frac{C_{it}}{C_{ox}}\right)\varphi_S\right) \equiv f_Q(V_{GS},\varphi_S)\,. \qquad \text{(S3-3)}$$

In Eq.(S3-3), C_{it} can be retrieved from the measured sub-threshold slope (S), using the relation $S=dV_{GS}/d\log(I_{DS})=kT/q\log(e)[1+C_{it}/C_{ox}]$ which can be derived from the diffusion current equation in the range $V_{FB}<V_{GS}<<V_T$. Here, $C_{it}=q^2D_{it}$ (where D_{it} is the interface state density).

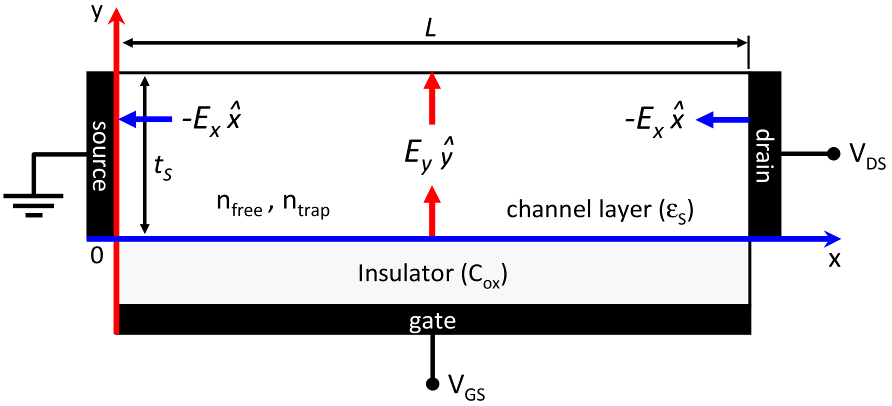

Figure S3.1: A schematic of a bottom gate thin film transistors (TFTs). Here, V_{DS} is drain voltage, V_{GS} is gate voltage, ε_S is the permittivity of channel layer, t_S is channel thickness, L is channel length, C_{ox} is gate insulator capacitance, and n_{free} and n_{trap} are free carrier and trapped carrier densities at localized tail and deep states (acceptor-like states), respectively.

S3.1.2. Mapping between Fermi Energy and Gate Voltage

To extract n_{free} as a function of V_{GS} from the transfer characteristics, the drain current equation when V_{DS}<<V_{GS} is as follows,

$$I_{DS}(V_{GS}) = \mu_0^* W Q_{free}(V_{GS}, V_{ch}(x)) \frac{dV_x}{dx}. \quad \text{(S3-4)}$$

Eq.(S3-4) can be approximated using the first order approximation at the source side (x=0): $dV_x/dx \approx V_{DS}/L$ with the initial conditions ($V_x(0)=0$ and $V_x(L)=V_{DS}$), where V_x is a channel potential along L. This can yield,

$$I_{DS}(V_{GS}) \approx \mu_0^* W Q_{free}(V_{GS}, 0)) \frac{V_{DS}}{L}, \quad \text{(S3-5)}$$

where W is the channel width. Also, Q_{free} can be represented by n_{free} and the effective thickness of free carrier sheet (λ_{free}) as follows,

$$Q_{free}(V_{GS}, 0) = q n_{free}(V_{GS}, 0) \lambda_{free}(V_{GS}, 0), \quad \text{(S3-6)}$$

where $\lambda_{free}(V_{GS},0)=[\varepsilon_S kT/q^2 n_{free}(V_{GS},0)]^{0.5}$.

From Eqs.(S3-5) and (S3-6), we have $n_{free}(V_{GS})$ at x=0 analytically represented as a function of V_{GS},

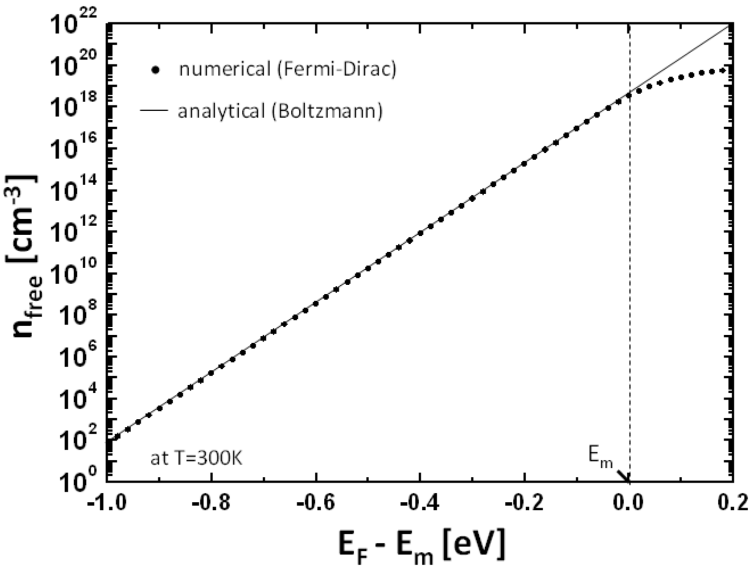

Figure S3.2: The computed free carrier density (n_{free}) at T=300K as a function of Fermi energy (E_F) from the conduction band minima (E_m), computed by analytical expression, derived from Boltzmann's approximation, in comparison with the numerical method using Fermi-Dirac statistics.

$$n_{free}(V_{GS},0) \approx \frac{I_{DS}^2(V_{GS})}{\varepsilon_S kT(\mu_0^*)^2(W/L)^2 V_{DS}^2} . \qquad \text{(S3-7)}$$

Based on Eq.(S3-7), the mapping between V_{GS} and φ_S can be computed with $n_{free}(\varphi_S)=N_C\exp[(E_{F0}-E_m+q\varphi_S)/kT]$ at x=0, assuming Boltzmann's approximation with $|E_{F0}-E_m+q\varphi_S| >> kT$. Note that E_{F0} can be determined by $E_{F0}-E_m \approx kT\cdot\ln[n_{free}(\varphi_S=0)/N_C]$. Now, we have an analytical expression of a mapping function ($f_M(V_{GS})$):

$$\varphi_S = \frac{2kT}{q}\ln\left(\frac{I_{DS}(V_{GS})}{\sqrt{N_C\varepsilon_S kT}\,\mu_0^*(W/L)V_{DS}}\right) + \frac{(E_C - E_{F0})}{q} \equiv f_M(V_{GS}) . \qquad \text{(S3-8)}$$

S3.2. Density of Localized Trap States

S3.2.1. Free and Trapped Carriers

First, n_{free} can be represented by the effective free carrier density (N_C), approximated from Boltzmann's approximation, kT<< (E_m–E_F),

$$n_{free}(E_F) = N_C \exp\left(\frac{E_F - E_m}{kT}\right). \quad \text{(S3-9)}$$

where N_C is defined as $2\left(2\pi m_n^* kT/h^2\right)^{3/2}$, where m_n^* is the effective mass of electrons and h is Planck's constant (6.63×10^{-34}J-s). For In-Ga-Zn-O, N_C is about 5×10^{18}cm^{-3} for m_n^*=0.34m_0 at T=300K, where m_0 is the electronic rest mass (9.11×10^{-31}kg). Fig.S3.2 shows the simulated results of n_{free} vs. E_F-E_m at T=300K, in comparison with the numerical results from Fermi-Dirac statistics, exhibiting a good agreement in the E_F range satisfied with (E_m–E_F)>>kT. Also, the trapped carrier density in the tail states can

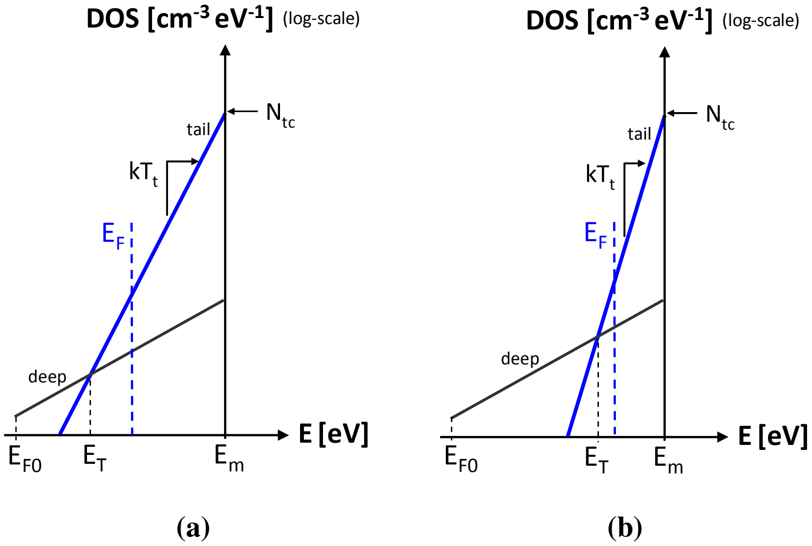

Figure S3.3: Exponential distribution of the tail states for (a) kT_t > kT and (b) kT_t< kT at a given ambient temperature (T) (e.g. T=300K, which is usually used as the reference ambient temperature for the analysis and modeling). The case (a) is related to the dispersive transport while trapping at tail states is negligible in the case (b). For both cases, Fermi level E_F is located within the tail states below E_m (E_F< E_m).

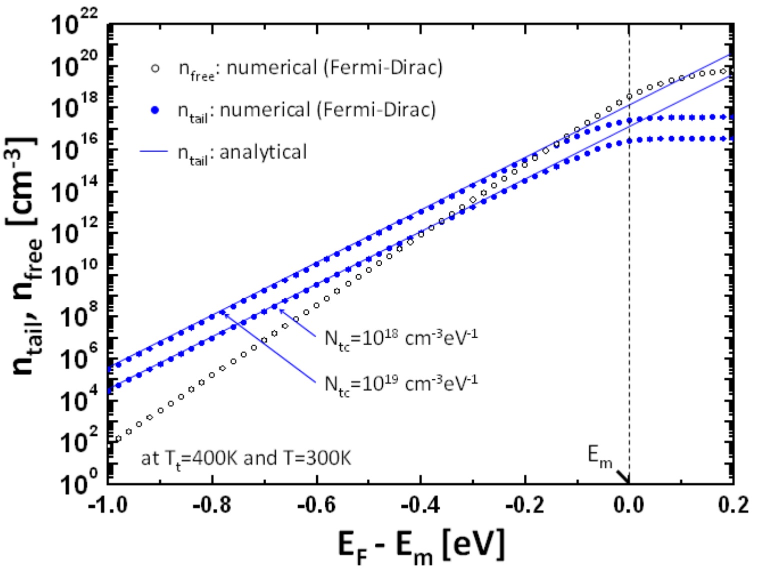

Figure S3.4: For an example case of $kT_t > kT$ (T_t=400K > T=300K), the computed trapped carrier density at tail states (n_{tail}) as a function of Fermi energy (E_F) from the conduction band minima (E_m), computed by analytical expression, Eq.(S3-12), approximated in this work, in comparison with numerical method using Fermi-Dirac statistics.

be defined as follows,

$$n_{tail} = \int_{E_{F0}}^{E_m} \frac{N_{tail}(E)}{1+\exp\left(\frac{E-E_F}{kT}\right)} dE \,. \tag{S3-10}$$

Based on Eq.(S3-10) can be rewritten with $u \equiv \exp\left(\frac{E-E_F}{kT_t}\right)$,

$$n_{tail}(E_F) = N_{tc} kT_t \exp\left(\frac{E_F - E_m}{kT_t}\right) f(u) \,. \tag{S3-11}$$

Here, we define $f(u) \equiv \int_0^{\exp\left(\frac{E_m - E_F}{kT}\right)} \frac{1}{1+u^{(T_t/T)}} du$. Depending on whether kT_t is larger than kT, Eq.(S3-11) yields a different solution. If a channel film has $kT_t > kT$, trapping at tail states are significant whereas the AOS has both cases depending on film quality (usually $kT_t < kT$). So, we need to consider both cases separately.

When kT_t > kT (T_t > T), $f(u) \approx \dfrac{\pi(T/T_t)}{\sin(\pi T/T_t)}$. Thus, Eq.(S3-11) can be represented analytically as follows,

$$n_{tail}(E_F) = N_{tc}\frac{\pi kT}{\sin(\pi T/T_t)}\exp\left(\frac{E_F - E_m}{kT_t}\right). \tag{S3-12}$$

If kT_t >> kT, $\sin(\pi kT/kT_t) \approx \pi kT/kT_t$. So, Eq.(S3-12) can be further simplified,

$$n_{tail}(E_F) \approx N_{tc}kT_t\exp\left(\frac{E_F - E_m}{kT_t}\right). \tag{S3-13}$$

Fig.S3.4 shows the simulated results of n_{tail} for kT_t>kT at T=300K for a different N_{tc} value, providing a good agreement with the numerical results in the E_F range satisfied with (E_m–E_F)>>kT. As shown in Fig.S3.4, n_{tail} has a less steep slope, determined by kT_t, than n_{free} with kT. So, it can be deduced that n_{tail} has a strong dependence on kT_t in terms of slope and temperature dependent behaviour. Additionally, as shown in Eq.(S3-13), n_{tail} for kT_t > kT is represented in terms of N_{tc}, kT_t, and the exponential term with kT_t.

When kT_t < kT (T_t < T), $f(u)$ can be solved as follows,

$$f(u) \approx \frac{1}{2}\exp\left(-\frac{E_F - E_m}{kT_t}\right)\left(\frac{2\ln\left(\exp\left(\frac{E_m - E_F}{kT_t}\right)+1\right)}{\exp\left(\frac{E_m - E_F}{kT_t}\right)}\right)^{(T_t/T)}. \tag{S3-14}$$

Also, Eq.(S3-14) can be further simplified with the condition that kT_t << kT,

$$f(u) \approx \frac{1}{2}\exp\left(-\frac{E_F - E_m}{kT_t}\right)\exp\left(\frac{E_F - E_m}{kT}\right). \tag{S3-15}$$

With (S3-15), Eq.(S3-11) yields for kT_t < kT,

$$n_{tail}(E_F) \approx \frac{1}{2}N_{tc}kT_t\left(\frac{2(E_m - E_F)}{kT_t}\right)^{(T_t/T)}\exp\left(\frac{E_F - E_m}{kT}\right). \tag{S3-16}$$

As can be seen in Eq.(3-11), n_{tail} for kT_t<kT is represented in terms of N_{tc}, kT_t,

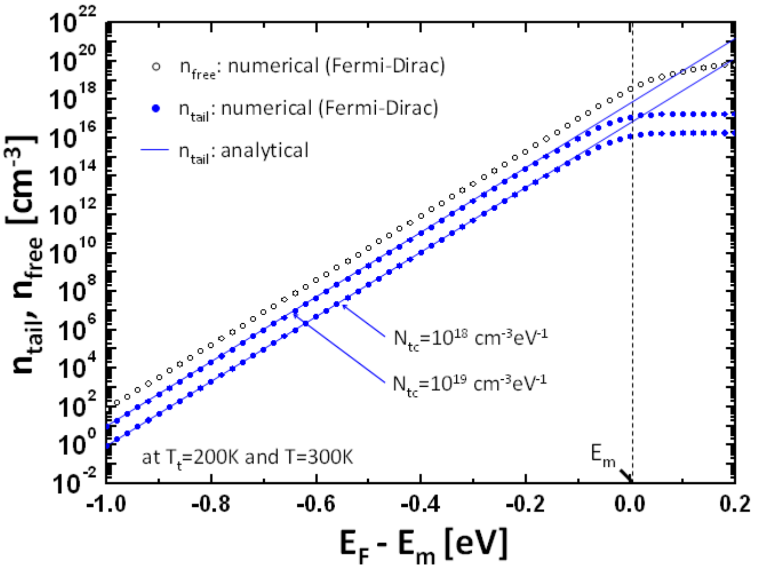

Figure S3.5: For the case of $kT_t<kT$ (T_t=200K < T=300K), the computed trapped carrier density at tail states (n_{tail}) as a function of Fermi energy (E_F) from the conduction band minima (E_m), computed by analytical expression, approximated in this work, in comparison with numerical method using Fermi-Dirac statistics.

and the exponential term with kT. Also, if $kT_t<<kT$, $\left(\frac{2(E_m-E_F)}{kT_t}\right)^{(T_t/T)}\approx 1$, thus $n_{tail}(E_F)\approx\frac{1}{2}N_{tc}kT_t\exp\left(\frac{E_F-E_m}{kT}\right)$. Interestingly, it is not dependent on the exponential term with kT_t. This is a big difference from the first case for $kT_t>kT$, as shown in Eq.(3-13). Fig.3.5 shows the simulated results of n_{tail} for $kT_t<kT$ at T=300K for a different N_{tc} value, providing a good agreement with the numerical results in the E_F range satisfied with $(E_m–E_F)>>kT$. Interestingly, n_{tail} has the same slope (kT) as n_{free} even if it has smaller kT_t, as shown in Fig.S3.5. This is the main difference from the first case with $kT_t>kT$.

The surface electric field E_S can be rewritten with Eq.(S3-2), applying Gauss's law (charge neutrality):

$$\sqrt{\frac{2q}{\varepsilon_S}\int_0^{\varphi_S}[n_{free}(\varphi)+n_{trap}(\varphi)]d\varphi} = \frac{Q_{free}+Q_{trap}}{\varepsilon_S}. \qquad \text{(S3-17)}$$

Using the first derivative of Eq.(S3-17) with respect to φ_S, Eq.(S3-17) can be represented as a function of φ_S, applying $Q_{free}+Q_{trap} = f_Q(V_{GS},\varphi_S)$ and $\varphi_S = f_M(V_{GS})$ from Eqs.(S3-3) & (S3-8), respectively:

$$n_{trap}(\varphi_S) = \frac{1}{2q\varepsilon_S}\frac{d}{d\varphi_S}\left[f_Q(f_M^{-1}(\varphi_S),\varphi_S)\right]^2 - n_{free}(\varphi_S). \qquad \text{(S3-18)}$$

Finally, the effective density of sub-gap states ($N_{sub\text{-}gap}$) as a function of Fermi energy (E_F) can be computed from the first derivative of Eq.(S3-18) with respect to φ_S:

$$N_{sub-gap}(\varphi_S) \approx \left.\frac{dn_{trap}(\varphi_S)}{d\varphi_S}\right|_{E_F\to E}, \qquad \text{(S3-19)}$$

where $\varphi_S = (E_F - E_{F0})/q$. Here, we assume that all filled traps are located below the Fermi energy, which corresponds to the condition $kT_t > kT$. In other words, the proposed method needs the hypothesis, $kT_t > kT$. Note that $N_{sub\text{-}gap}(E) = N_{nc}\exp[(E-E_m)/kT_n]$ (where n denotes t or d) is the same as the first derivative of Eq.(S3-13), replacing E_F with E for $N_{sub\text{-}gap}(E)$. Thus, only for $kT_t > kT$, the first derivative of $n_{trap}(E_F)$ allows us to find the $N_{sub\text{-}gap}(E)$.
